# Plant phosphomannose isomerase as a selectable marker for rice transformation

**DOI:** 10.1038/srep25921

**Published:** 2016-05-13

**Authors:** Lei Hu, Hao Li, Ruiying Qin, Rongfang Xu, Juan Li, Li Li, Pengcheng Wei, Jianbo Yang

**Affiliations:** 1 Key Laboratory of Rice Genetic Breeding of Anhui Province, Rice Research Institute, Anhui Academy of Agricultural Sciences, Hefei, 230031, China; 2 School of Agronomy, Anhui Agricultural University, Hefei, 230036, China

## Abstract

The *E. coli phosphomannose isomerase (EcPMI*) gene is widely used as a selectable marker gene (SMG) in mannose (Man) selection-based plant transformation. Although some plant species exhibit significant PMI activity and active PMIs were even identified in Man-sensitive plants, whether plant *PMIs* can be used as SMGs remains unclear. In this study, we isolated four novel *PMI* genes from *Chlorella variabilis* and *Oryza sativa*. Their isoenzymatic activities were examined *in vitro* and compared with that of EcPMI. The active plant *PMIs* were separately constructed into binary vectors as SMGs and then transformed into rice *via Agrobacterium*. In both *Indica* and *Japonica* subspecies, our results indicated that the plant *PMIs* could select and produce transgenic plants in a pattern similar to that of *EcPMI*. The transgenic plants exhibited an accumulation of plant *PMI* transcripts and enhancement of the *in vivo* PMI activity. Furthermore, a gene of interest was successfully transformed into rice using the plant *PMIs* as SMGs. Thus, novel SMGs for Man selection were isolated from plants, and our analysis suggested that *PMIs* encoding active enzymes might be common in plants and could potentially be used as appropriate genetic elements in cisgenesis engineering.

During plant transformation, transformed cells are identified from a matrix consisting primarily of untransformed cells using a selection system and then regenerated to transgenic plants. Numerous selection systems have been developed in the past 20 years; nevertheless, only a few of them have been practically used^1^,^2^. The most-used selection systems consist of an antibiotic or herbicide selective agent, which is toxic for the non-transformed cells, and the corresponding selectable marker gene (SMG) confers the tolerance to transformed cells^3^. These systems are stable and efficient in both scientific research and commercial breeding. However, relevant safety concerns have also been continually raised by the public and by regulatory committees in certain countries. One concern is that the use of toxic selection agents in factories and fields has potential risks to the environment. Another concern is the safety risks of SMGs. Because bacteria can import and integrate exogenous DNA^4^,^5^, the natural bacterial population may be exposed to recombinant DNA, including SMG fragments, during the cultivation and consumption of genetically modified (GM) crops and therefore lead to the possibility of unintended horizontal gene transfer (HGT)^6^,^7^. HGT may spread antibiotic resistance from the SMGs of GM plants to soil or gastrointestinal tract microbes, which may compromise the clinical effectiveness of antibiotic drugs. Another risk is caused by the gene flow between GM plants and their wild relatives^8^. Because SMGs do not naturally exist in plants, the spread of novel antibiotic and herbicide resistance traits may lead to the extinction of wild populations^9^ or to increased fitness and persistence of wild relatives, potentially making them “super-weeds”^10^. Moreover, antibiotic or herbicide SMGs are generally isolated from bacteria^3^ and thus are cited as “unnatural”. Although the biological safety of SMGs for human and animal health has been validated by several independent risk-assessment tests^11^,^12^,^13^, their bacterial origins are easily associated with potential danger, causing rejection by consumers.

Rice is the most preferred staple food in Asia and a major target for plant genetic engineering. It has been more than a quarter century since the successful genetic transformation of rice. Countless studies have revealed that transgenes can effectively improve the yield, development, nutrition, stress resistance and other agronomic traits of cultivated rice varieties. However, even under strict monitoring, few types of GM rice have been commercially released to date^14^. Safety concerns regarding SMGs are major deterrents to taking advantage of the advancement of biotechnology in rice breeding. Several approaches, including co-transformation systems, recombination-mediated site-specific excision or integration systems, and even CRISPR-based targeted degradation systems, have been designed and/or applied to generate marker-free transgenic plants^15^,^16^,^17^,^18^. However, these approaches normally require the screening of a large number of transformation events or extra transformation steps, which would greatly increase labor and time costs. One alternative strategy is the application of a non-antibiotic and non-herbicide selection system during transformation. A *phosphomannose isomerase (PMI*)/mannose (Man) combination is one of the most popular and highly used non-antibiotic systems in rice. Man itself is not toxic to plants; however, Man is phosphorylated to mannose-6-phosphate (M-6-P) by endogenous hexokinases. The accumulation of M-6-P in plants blocks glycolysis^19^, inhibits ATP production^19^,^20^, and represses photosynthetic gene transcription^21^. Consequently, plant cells cultivated in Man media grow much slower, and organogenesis and germination are strongly restrained^22^,^23^. PMI can convert M-6-P into fructose-6-phosphate (F-6-P), which is then metabolized *via* glycolysis in plants. Therefore, only cells expressing *PMI* can grow and be selected on medium that uses Man as a carbon source, which enables the use of *PMI* as a SMG in plant transgenes.

PMIs are widely found in bacteria, fungal and animal cells; however, PMIs were commonly considered nonexistent in higher plants, except in some soybean species^24^,^25^. Recently, a few active *PMIs* were identified in *Arabidopsis* and Chinese cabbage^26^,^27^, suggesting that functional *PMI* may exist in higher plants. The existence of *PMI* isoforms in plant genomes consequently raises a question: Are the higher plant-originated *PMI* genes sufficiently active to be SMGs for Man selection of plant transgenes? Here, we report that four novel *PMIs* were isolated from algae and rice. The corresponding proteins were expressed and purified from *E. coli*, and their enzymatic activities were identified. Furthermore, the active plant *PMI* genes were expressed in cells to select and regenerate transgenic rice under Man pressure. Our results suggested that plant *PMIs* could be applied as effective alternative SMGs in the genetic engineering of rice and other plants.

## Results

### Cloning of the plant *PMIs* and characterization of the sequences

The genomic sequence databases of *Chlorella variabilis* and *Oryza sativa* were searched to identify plant *PMIs*. Four putative *PMIs* were found, including a gene from *Chlorella (CHLNCDRAFT_139231*, designated as *CvPMI*) and three genes from rice (*Os01g0127900, Os09g0389000* and *Os11g0600900*, designated as *OsPMI1, OsPMI2* and *OsPMI3*, respectively). The coding sequences of the *PMIs* were cloned, and the predicted molecular masses for CvPMI, OsPMI1, OsPMI2 and OsPMI3 were 45.85, 46.93, 44.18 and 24.15 kDa, respectively. The alignment of amino acid sequences revealed that the sequences of these plant PMIs were highly similar to the classic type I PMIs of *C. albicans, E. coli* and humans (Fig. S1). As indicated in Fig. S1, all plant PMIs except OsPMI3 contained the eukaryotic PMI consensus sequence Y*X*D*X*NHKPE in their primary structures^28^. Type I PMI is a zinc-dependent metalloenzyme. Four amino acids of *C. albicans* PMI (CaPMI) (Gln-111, Glu-138, His-113 and His-285) are essential for the ligand of zinc^29^. These residues were also conserved in the sequences of the plant PMIs except OsPMI3 (Fig. S1). Furthermore, the three-dimensional (3D) structures of the PMIs were predicted. Except for OsPMI3, these plant PMIs were likely to exhibit a structure highly similar to that of CaPMI (Fig. S2), suggesting that most plant PMIs may have the same biochemical activity as the typical type I PMI.

### Identification of plant PMI isoenzymatic activity

**Figure 1:**
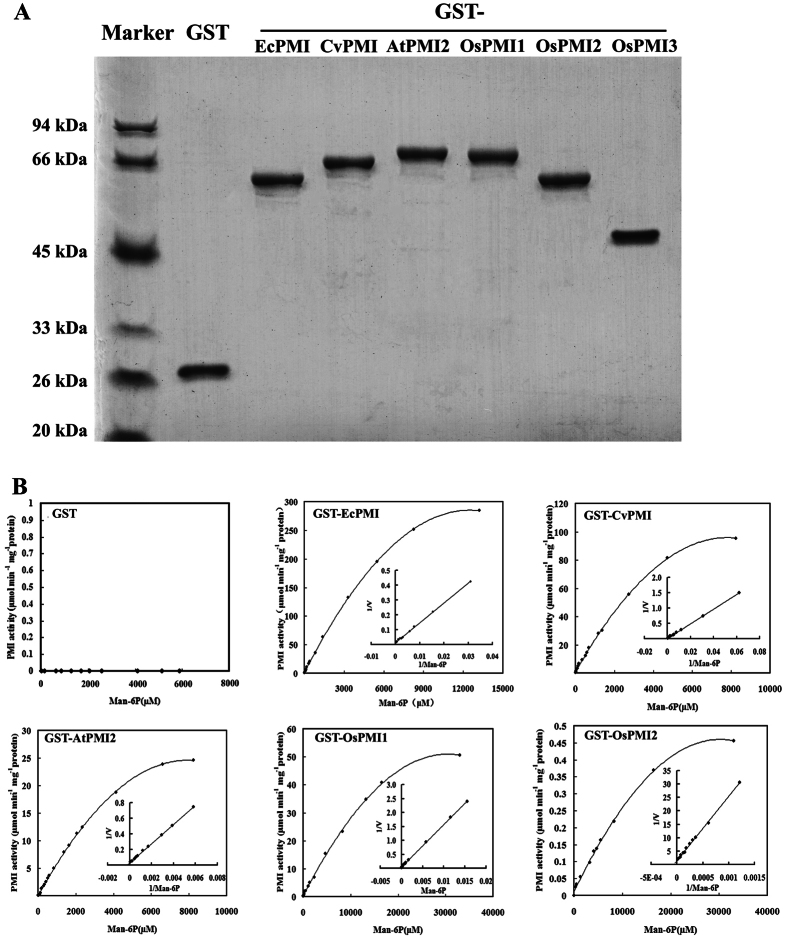
Identification of isoenzymatic activities of PMIs *in vitro*. (**A**) Purified GST-tagged recombinant PMI proteins. The recombinant proteins and GST tag were expressed in *E. coli*, purified by affinity chromatography, and verified using SDS-PAGE with Coomassie Blue staining. Each line contains 2 μg of the respective recombination protein. (**B**) Affinities of recombinant PMI proteins for M-6-P. The isomerization reactions were monitored at 30 °C by a coupled enzyme method.

To determine whether plant PMIs have the ability to isomerize M-6-P and to estimate whether their activities are sufficient to be used as potential SMGs, the four PMIs were fused with a glutathione S-transferase (GST) tag at the N-terminal and expressed in *E. coli* cells. Two previously identified PMIs, EcPMI from *E. coli* and AtPMI2 from *Arabidopsis*, were expressed as controls. Five GST-PMI fusion proteins were purified from the soluble fraction of bacterium to homogeneity using affinity purification. Although many expression conditions had been tested, GST-OsPMI2 was not soluble. Therefore, the protein was denatured in inclusion bodies and subsequently recovered and purified. The purity of the recombined proteins was assessed *via* sodium dodecyl sulfate-polyacrylamide gel electrophoresis (SDS-PAGE) analysis (Fig. 1A).

To quantitatively evaluate the isoenzymatic activity *in vitro*, the values of *K*m and *V*max for the M-6-P to F-6-P isomerization reaction catalyzed by the expressed fusion proteins were measured by the phosphoglucose isomerase (PGI)/D-glucose-6-phosphate dehydrogenase (G6PDH)-coupled enzyme method^26^. As expected, the GST tag itself did not convert M-6-P (Table 1). Accordingly, the activity of the fusion proteins should be contributed by the corresponding PMIs. As shown in Fig. 1B and Table 1, GST-CvPMI had the highest *V*max (68.03 ± 2.22 μmol min^−1^ mg^−1^ protein) among the plant PMIs, which was comparable with that of EcPMI (74.63 ± 5.34 μmol min^−1^ mg^−1^ protein). However, the *K*m value of CvPMI was greater than that of EcPMI, suggesting lower substrate affinity of the recombinant CvPMI proteins. The *K*m and *V*max of GST-AtPMI2 were 4104.67 ± 53.47 μM and 33.33 ± 2.42 μmol min^−1^ mg^−1^ protein, respectively. In a previous study, the *K*m and *V*max parameters of the histidine-tagged AtPMI2 were determined to be 372 ± 13 μM and 22.5 μmol min^−1^ mg^−1^ protein, respectively^26^. The differences between the kinetic parameters may be due to the different environments of the enzymatic assays and/or to interference from the protein tags. The parameters of OsPMI1 were similar to those of AtPMI2, implying that they may have similar biochemical activity. Moreover, the activities of OsPMI2 were much lower than were those of OsPMI1 possibly due to denaturation during the protein purification process. Furthermore, the activities of OsPMI3 were not detected, which is consistent with the lack of the conserved type I PMI motif in its sequence.

### Transformation of *Japonica* and *Indica* rice using three plant *PMIs* as SMGs

**Figure 2:**
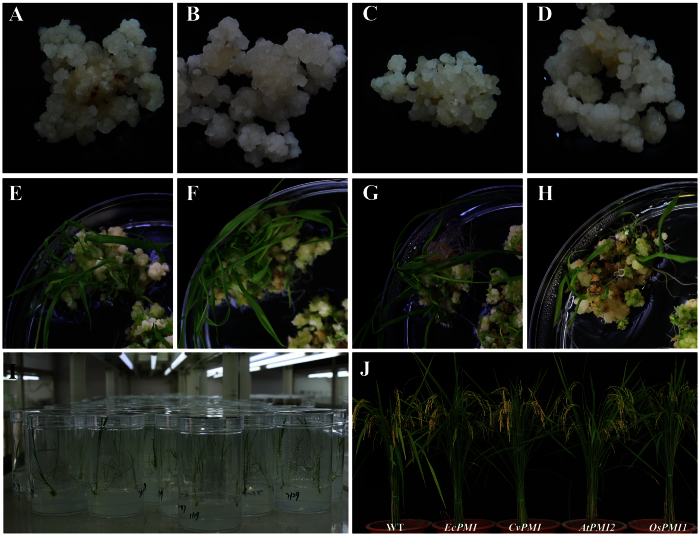
The transformation of Nipponbare using different *PMI*/Man systems. The resistant calli of Nipponbare were selected by Man after incubation with *Agrobacterium* vectors using *EcPMI* (**A**), *CvPMI* (**B**), *AtPMI2* (**C**) and *OsPMI1* (**D**) as SMGs. The shoots regenerated from the resistant calli generated by the vectors using *EcPMI* (**E**), *CvPMI* (**F**), *AtPMI2* (**G**) and *OsPMI1* (**H**) as SMGs. Then, roots were grown in the rooting medium (**I**). The transgenic Nipponbare harboring the *PMI* vectors grew and developed normally in the greenhouse. From left to right: the untransformed plant (WT), the transgenic plants harboring the derivate pCAMBIA 1381 vector with *HPT* replaced with *EcPMI, CvPMI, AtPMI2* and *OsPMI1* and selected by Man.

To assess whether plant *PMIs* could potentially function as SMGs for rice transformation, the three highly active *PMIs (CvPMI, OsPMI1* and *AtPMI2*) were inserted into a pCAMBIA1381 binary vector under the control of the CaMV 35S promoter (35S) to replace the *hygromycin phosphotransferase (HPT*) SMG. A similar vector using *EcPMI* substituted for *HPT* was constructed in parallel and used as the positive control. Previously, we optimized a high-throughput *Agrobacterium*-mediated rice transformation system for *EcPMI* selection^30^. In this study, the selection abilities of the three plant *PMIs* were initially examined in the *Japonica* cultivar Nipponbare following the same protocol. Similar to the effect of *EcPMI*, resistant calli grew from the calli transformed with the *CvPMI, OsPMI1* and *AtPMI2* vectors under selection with 12.5 g/L Man plus 5 g/L sucrose (Fig. 2A–D). The selection rates of *CvPMI* were comparable with those of *EcPMI* and were slightly higher than the selection rates of *OsPMI1* and *AtPMI2* (Table 2). The selection of the *PMIs* was also tested in an *Indica* variety, Kasalath. Following the *Indica* transformation protocol of *EcPMI* constructs, 7.5 g/L Man plus 15 g/L maltose were added to the medium as selection pressure^31^. Similar to the results in Nipponbare, the resistant calli were developed after being transformed with the *PMIs*, and the selection rates of *CvPMI* were even higher compared with those of *EcPMI* (Table 2). These results preliminarily suggested that the plant *PMIs* have the ability to be used as SMGs in rice transformation.

Next, the resistant calli selected by the *PMI* SMGs were tested for regeneration. In both Nipponbare and Kasalath, plantlets were obtained (Fig. 2E–H), and the plant *PMIs* exhibit an efficiency comparable to that of *EcPMI* (Tables 2 and S1–S2). Then, a regenerated plantlet was transformed in rooting medium (Fig. 2I) and confirmed by sequence-specific PCR analyses. As indicated in Tables 2 and S1–S2, *CvPMI* provided transformation frequencies (TFs) similar to those of *EcPMI* in Nipponbare and much higher than those in Kasalath. Although *AtPMI2* and *OsPMI1* were less efficient than *EcPMI* in Nipponbare, their TFs were close to that of *EcPMI* in Kasalath. These results suggested that transgenic rice could be efficiently generated using the plant *PMIs* in a manner similar to *EcPMI*. Furthermore, the low-copy rates in transgenic events were investigated by a TaqMan assay of the 35S promoter. The construct with a reduced low-copy rate was believed to produce insufficient PMI activity and was not recommended for rice transformation^31^. However, low-copy rates varied significantly between independent experiments (Tables S1–S2), suggesting that the rates may also be related to reasons other than the constructs or SMG activity.

The low-copy transgenic plants were transferred into soil, and no significant abnormality in growth or development was observed in the transgenic population of Nipponbare or Kasalath (Figs 2J and S3). The seeds of the T_0_ plants were germinated on Man selection medium to further examine the selection effect of the plant *PMIs*. The germination of untransformed rice seeds was completely inhibited on the MS medium plus 12.5 g/L Man. By contrast, most seeds of the plant *PMI* transgenic lines could germinate and grow into plants (Fig. S4). These data further confirmed that the plant *PMIs* could be used to select the positive transgenic lines in rice transformation.

### Optimization of regeneration medium for plant *PMIs*

The escape of untransformed cells under Man selection frequently occurs when using the *EcPMI* SMG, as noted in previous reports, as well as when using the plant *PMI* SMGs in this study. One possible reason might be reduced Man pressure in the regeneration medium. Therefore, we tested the escape rate and regeneration rate of the three plant *PMIs* and *EcPMI* under different Man concentrations in the corresponding Nipponbare transformation. In general, the regeneration rates decreased as Man pressure increased, and escape was remarkably avoided by Man supplementation of the regeneration medium, as expected (Tables 3 and S3). In particular, the regeneration rates of the *AtPMI2* and *OsPMI1* transformants were similar to or slightly lower than that of *EcPMI*. Their transformants produced few plants or did not produce any plant under additional selection pressure in the regeneration medium (Table 3). However, the regeneration rate of the *CvPMI* transformants was much higher under Man pressure compared with *EcPMI*, and approximately 12.5% of plants could still be produced even in medium using Man as sole carbon source (Table 3).

### Expression of PMIs in the corresponding transgenic plants

**Figure 3:**
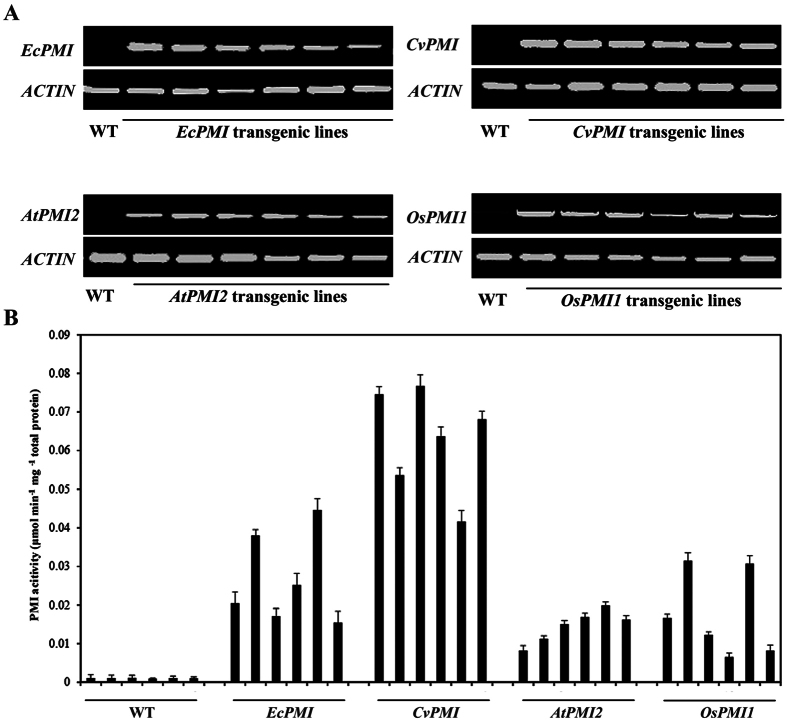
Identification of the expression of *PMIs* in transgenic plants. (**A**) Semi-quantitative RT-PCR analysis of the expression of the *PMIs*. Total RNA was prepared from seedlings of untransformed rice (WT) and independent low-copy transgenic lines at 10 days after germination (DAG) and reverse-transcribed to cDNA. RT-PCR was carried out using sequence-specific primers for respective *PMIs*, and *OsACTIN1* was used as an internal control. (**B**) Quantitative analysis of total PMI activity of rice leaves. The activity was determined in 10-DAG rice leaves of untransformed rice (WT) plants as a control. For transgenic plants, six independent low-copy T_1_ lines of each *PMI* construct were randomly selected and examined. The plants were germinated under Man selection, and the leaves of 10-DAG resistant plants were collected to extract total proteins and to determine the isoenzymatic activity. The presented values are the mean ± SD of three technical replicates.

To further verify the transgenic plants selected by the *PMI*/Man system, reverse-transcription PCR (RT-PCR) was performed to examine the expression of the *PMIs* in the corresponding transgenic lines of Nipponbare. According to the semi-quantitative RT-PCR results, the exogenous *CvPMI* and *AtPMI2* genes were expressed in the corresponding regenerated plants, and the abundance of the *OsPMI1* transcript greatly increased (Fig. 3A). In addition, the PMI activities of the plants were examined in six independent low-copy transgenic lines of each construct and untransformed plants. As expected, the PMI activities of the proteins isolated from transgenic plants were enhanced (Fig. 3B). In total, 14.70- to 42.44-fold, 39.60- to 70.98-fold, 7.82- to 19.00-fold and 6.27- to 30.02-fold increases in the PMI activities of the *EcPMI, CvPMI, AtPMI2* and *OsPMI1* transgenic plants, respectively, were noted compared with untransformed rice. These data suggest that the overexpression of *CvPMI, AtPMI2* and *OsPMI1* should enhance the PMI activity of rice cells; therefore, these genes are effective as SMGs for Man selection.

### Transformation of a gene of interest (GOI) in rice using the plant *PMI*/Man system

To assess whether plant *PMIs* could be used as SMGs for GOI transformation, three binary vectors that contained both the maize ubiquitin 1 promoter (*Ubq*)-driven individual plant *PMI* cassette (the SMG) and the 35S-driven *HPT* cassette (GOI) were constructed. The transformation of the corresponding *PMI*-*HPT* vectors into Nipponbare was similar to that of the above-described vectors containing each SMG alone. As listed in Table 4, no obvious difference in the selection and regeneration efficiency of the sole SMG vector and the corresponding SMG-GOI vector was observed. GOI transformations in the regenerated plants were molecularly identified by sequence-specific PCR of the *PMI* cassette and the *HPT* cassette (the *Ubq* promoter and *HPT* gene, respectively), and almost all *PMI*-positive plants had an *HPT* fragment (Table S4). We noticed that plants lacking *HPT* also lacked a *PMI* cassette, suggesting that the regenerated plants lacking the GOI may be caused by the escape of cells under Man pressure. Furthermore, six T_0_ lines of each construct were randomly selected, and their seeds were collected and germinated under Man and hygromycin selection separately. As expected, the T_1_ population could resist both selection agents, and the progeny segregation of Man resistance was similar to that of hygromycin resistance (Table S5). Taken together, these data suggested that the GOI could be reliably and efficiently transformed into rice by the plant *PMI*/Man system.

## Discussion

All current *PMI*/Man systems use *EcPMI* as the SMG, except for a few examples that take advantage of the yeast *PMI*. Apparently, there are significant differences in the gene expression between prokaryotes or yeast and higher plants. In a previous study, we showed that the utilization of the plant codon-optimized *EcPMI* exhibited higher selection efficiency in rice transformation, which suggested that the expression of the original *EcPMI* may be partially inhibited in plants^32^. Successful applications of the *PMI* system are dependent on the plant’s inability to utilize Man. The disappearance of PMI activity in plants was long presumed to stem from the absence of the *PMI* gene in the genome^33^. However, genome research has revealed that the homologous genes of type I *PMI* are universally observed in higher plants^27^. One conventional explanation was that the plant homologies of *PMI* might be deficient in isoenzymatic activity. In contrast to this hypothesis, biochemical assay of the histidine-tagged AtPMI1 and AtPMI2 proteins produced in *E. coli* indicated that these enzymes are activated *in vitro*^26^. The function of *AtPMI2* was also examined in our study. The purified GST-AtPMI2 protein exhibited obvious M-6-P catalytic activity *in vitro*. In addition, *AtPMI2* overexpression significantly enhanced the *in vivo* PMI activity in rice. Both of these results further confirmed that the AtPMI2 protein should be an active isoenzyme. Rice is a typical plant that is sensitive to Man accumulation^34^,^35^. OsPMI1 has a conserved sequence motif of the typical PMIs. Similar to AtPMI2, GST-OsPMI1 clearly displayed PMI activity. Although the activities of AtPMI2 and OsPMI1 were lower than that of EcPMI *in vitro*, their selection efficiencies of rice transformation were comparable. Additionally, the proteins of the transgenic plants expressing *AtPMI2, OsPMI1* and *EcPMI* exhibited the same levels of biochemical activity regarding M-6-P conversion. These data imply that the *in vivo* activities of AtPMI2 and OsPMI1 may be similar to that of EcPMI. Because *AtPMI2* and *OsPMI1* also provide sufficient ability to metabolize Man in plants, we hypothesize that these two *PMI*s can be used as potential substitutes of *EcPMI* in the existing *PMI*/Man selection system. In addition, our results indicate that rice overexpressing *OsPMI1* can grow under Man pressure, which suggests that the absence of PMI activity in rice should be caused by extremely low *OsPMIs* expression. Notably, background PMI activity can be detected in many Man-sensitive plants, such as *Arabidopsis*, papaya, sugarcane and Chinese cabbage^36^,^37^,^38^, and putative type I *PMIs* do exist in the genomes of plants. Therefore, we hypothesize that the overexpression of *PMIs* of Man-sensitive plants may also enhance the PMI activity of cells and thereby may be utilized as alternative SMGs in plant genetic engineering.

Earlier reports suggested that most plants lack PMI or have very low PMI activity^39^, whereas a few species with high activity were noted in subsequent studies. Man is deeply involved in the metabolism of mannitol^40^. In some legumes and umbelliferous plants (such as *Cassia coluteoides* and celery), mannitol is the major photosynthetic product and/or translocated carbohydrate. Unsurprisingly, these plants have high levels of PMI^41^,^42^. Other plants, including certain algae as well as some varieties of grape and sweet potato, can easily overcome the growth toxic effect of high concentrations of exogenous Man and can even use Man as an exclusive carbon source during growth^43^,^44^,^45^. Although direct evidence is lacking, it is reasonable to presume that their PMIs should be highly active. In this study, CvPMI, which was encoded by the only copy of *PMI* in the genome of the Man-tolerant *Chlorella*, exhibited a strong activity *in vitro*. The PMI activities in most *CvPMI*-overexpressing plants were higher than were those in transgenic plants carrying *EcPMI, AtPMI2* and *OsPMI1* overexpression constructs. Although the protein levels of PMIs might differ in these plants, we could still infer that CvPMI was more active *in vivo* than EcPMI or the PMIs isolated from Man-sensitive plants because they were driven by the same promoter. In addition, rice cells carrying *CvPMI* regenerated more effectively than the other *PMIs* under a high concentration of Man, implying that *CvPMI* could confer stronger Man metabolism than *EcPMI* and the other plant *PMIs*. Taken together, our results suggest that the *PMI* homologs from Man-tolerant plants might be used as better SMGs than *EcPMI* in transformation systems of rice and other plants.

Because Man itself does not kill non-transformed cells, the positive selection of Man normally has a higher efficiency than the negative selection of antibiotic or herbicide systems. We showed that three plant *PMIs* all exhibited high selection efficiencies in *Indica* and *Japonica* in this study. Using the same binary vectors containing the plant *PMI* expression cassette and the *HPT* cassette, Man selection is much more efficient than hygromycin selection (*HPT* selection data are indicated in Table S6). The escape issue is much more serious in *PMI*/Man systems. Given that Man seriously affects plant organogenesis, the regeneration medium of the conventional rice transformation protocol using *EcPMI* normally contains no or a low concentration of Man. Generally, approximately 5% to 10% of regenerated plants (depending on constructs, transformation protocols and rice varieties) produced by *EcPMI*/Man systems are non-transgenic plants^30^,^31^,^34^,^46^. Our data indicate that *CvPMI* provided more efficient regeneration compared with *EcPMI* under a high concentration of Man and that the escape rates were greatly reduced with the increase in Man. These data suggest that the combination of optimized Man medium and *CvPMI* or other highly active plant *PMIs* may overcome the escape issue of *PMI*/Man systems.

The social concern of consuming GM crops is partly attributed to the notion of eating “unnatural” DNA and its products. The safety of *EcPMI* has been well evaluated by a series of risk assessments, and no adverse effects on the health of mammals or on the environment have been demonstrated^47^,^48^. However, this protein remains a “foreign” element in transgenic plants. We showed that at least three plant *PMIs* were sufficiently powerful SMGs for rice transformation, suggesting that plant *PMIs* can be used as substitutes of *EcPMI* in plant genetic engineering. According to the principle of cisgenesis^49^,^50^,^51^, we believe that plant transformation systems using their own *PMI* or *PMIs* from closely related varieties could potentially alleviate the skepticism of the public regarding transgenic technology in plants.

## Methods

### Gene cloning and vector construction

To clone the plant *PMIs*, total RNA was isolated from a culture of *Chlorella variabilis* NC64A, mature leaves of *Oryza sativa* L. ssp. *japonica* cv. Nipponbare and seedlings of *Arabidopsis thaliana* using an RNAprep pure Plant Kit (Tiangen, China). Then, RNA was reverse-transcribed to cDNA, which served as the PCR template. The coding sequences (CDSs) of the *PMIs* were amplified by a high-fidelity FastPfu Fly DNA polymerase (TransGen Biotech, China) and confirmed by sequencing. To construct the vectors for recombinant protein expression, the CDSs of *PMIs* were inserted into the pGEM-4 T-1 vector by double enzymatic digestion. To construct the binary vectors for rice transformation, the *PMIs* replaced the region of *HPT* in the pCAMBIA 1381 by *Xho* I digestion. All primers used in this study are listed in Table S7.

### PMI expression and GST affinity purification

The empty and *PMI*-inserted pGEM-4 T-1 vectors were transformed into the *E. coli* strain *Rosetta (DE3) plysS* to express recombinant proteins. A 5-mL overnight liquid culture was used to inoculate 500 mL of lysogeny broth (LB) in 1-L Erlenmeyer flasks, and the cultures were grown at 37 °C until OD_600_ = 0.6 ~ 0.8. Then, induction with isopropyl-β-D-1-thiogalactopyranoside (IPTG) was performed at a final concentration of 1 mM. The cultures were then grown for an additional 20 h at 18 °C before harvesting. The cell pellet was suspended, washed and re-suspended with 10 mL of PBS buffer (pH 7.4) containing 1 mM phenylmethyl sulfonyl fluoride (PMSF), 1 mg/mL lysozyme and 1% protease inhibitor cocktails (AMRESCO, USA). Cell disruption was achieved by sonic vibration in an ultrasonic processor and centrifuged at 12000 rpm for 15 min to remove debris. The supernatants were then incubated with GST resin (GE Healthcare, USA) for 2 h, loaded onto a purification column and sequentially eluted with PBS buffer, low salt buffer (50 mM Tris-HCl, 10 mM reduced glutathione, pH 8.0) and high salt buffer (50 mM Tris-HCl, 20 mM reduced glutathione, 150 mM NaCl, pH 8.0) according to the standard protocol of the manufacturer. The recombinant proteins were desalted and concentrated by centrifugal filter devices (Millipore, USA).

### PMI *in vitro* assay

The *in vitro* activity of PMI was measured at 30 °C using a coupled enzymatic assay based on a modified protocol^52^. The 300 μL total volume reaction mixture contained 257 μL of Tris-HCl buffer (50 mM, pH 7.5), 25 μL of D-M6P (over 10 concentrations ranging from 4 μM to 48000 μM, Sigma), 10 μL of NADP (13.5 mM, Sigma), 2 μL of phosphoglucoisomerase (PGI, 1 kU/mL, Sigma) and 1 μL of glucose 6-phosphate dehydrogenase (G6PDH, 1 kU/mL, Sigma). The mixture was equilibrated to 30 °C, and the absorbance was monitored at 340 nm (A_340_) until constant. Then, 5 μL of diluted extract was added to initiate the reaction. The increase in A_340_ was recorded for approximately 10 min. Enzyme activities were calculated from the initial linear rates of cofactor reduction after the subtraction of endogenous activities (which were measured in assays without substrate) in a Lineweaver-Burk assay. The protein concentration of extracts was estimated by the Bradford method, with bovine serum albumin (BSA) as the protein standard. Enzyme assays were performed on freshly prepared extracts, and each value was an average of three experiments. One unit of enzymatic activity converted 1 μmol of D-M6P to D-F6P per min.

### PMI *in vivo* assay

The specific activity of PMI *in vivo* was determined according to the coupled enzymatic assay procedure, with a slight modification^53^. Young leaf tissues (500 mg) were extracted in 400 μL of ice-cold Tris-HCl (50 mM, pH 7.5) containing 1% protease inhibitor cocktail (Sigma), 1 mM phenylmethylsulfonyl fluoride (PMSF), 1 mM dithiothreitol (DTT) and 1 mM polyvinylpyrrolidone (PVP). One hundred microliters of crude protein extract was added to the substrate solution consisting of 75 μL of NADP (13.5 mM), 1 μL of PGI (1 kU/mL), and 0.5 μL of GDPDH (1 kU/mL) and incubated for 30 min at 37 °C to remove any endogenous D-M6P. The reaction was initiated by adding 20 μL of D-M6P (50 mM), and the ▵A_340_ was recorded for 60 min to calculate the activities.

### Rice transformations

The transformations followed previously described protocols^30^,^31^ with modifications. The embryos were isolated from mature rice (Nipponbare and Kasalath) seeds, and explants were induced at 30 °C for two weeks. The *Agrobacterium*-incubated calli were transferred to the recovery medium containing 250 mg/L carbenicillin for five days and then selected on Man-containing media for three weeks. Calli on the selection media were counted, and the resistant calli that emerged from each callus were regarded as one event. Then, two to three callus nodules from one resistance event were transformed in the regeneration medium for three weeks. Only one shoot from each event was transferred to the rooting medium. Then, the plants grew and produced seeds in a greenhouse. The medium compositions were identical to previously reported recipes^30^,^31^. All chemical agents used in the rice transformation were obtained from Sigma-Aldrich Co., USA.

## Additional Information

**How to cite this article**: Hu, L. *et al*. Plant phosphomannose isomerase as a selectable marker for rice transformation. *Sci. Rep.* **6**, 25921; doi: 10.1038/srep25921 (2016).

## Supplementary Material

Supplementary Information

## Figures and Tables

**Table 1:** PMI activities of the GST-fused protein evaluated by the PGI/G6PDH-coupled method.

Proteins	*K*m (μM)	*V*max (μmol min^−1^mg^−1^protein)
GST	*N.D.*	*N.D.*
GST-EcPMI	999.18 ± 7.38	74.63 ± 5.34
GST-AtPMI2	4104.67 ± 53.47	33.33 ± 2.42
GST-CvPMI	1625.44 ± 19.58	68.03 ± 2.22
GST-OsPMI1	3808.98 ± 29.65	24.94 ± 3.17
GST-OsPMI2	6462.27 ± 47.93	0.33 ± 0.04
GST-OsPMI3	*N.D.*	*N.D.*

*N.D.*: not detected.

**Table 2:** Comparison of rice transformation using *EcPMI* and plant *PMIs* as SMGs.

SMG^a^	Selection rate (%)^b^		Regeneration rate (%)^c^		Positive rate (%)^d^		Transformation frequency (%)^e^		Low-copy rate (%)^f^	
	*Nip.*	*Kasa.*	*Nip.*	*Kasa.*	*Nip.*	*Kasa.*	*Nip.*	*Kasa.*	*Nip.*	*Kasa.*
*EcPMI*	63.22 ± 1.22	43.71 ± 5.38	69.71 ± 1.32	83.66 ± 0.69	91.88 ± 1.32	92.44 ± 1.37	40.53 ± 2.13	33.71 ± 3.38	27.08 ± 2.08	27.39 ± 7.78
*CvPMI*	57.00 ± 6.33	59.23 ± 2.31	72.63 ± 3.16	81.13 ± 4.37	89.87 ± 0.28	94.08 ± 1.83	37.03 ± 2.63	45.38 ± 5.08	27.01 ± 9.96	43.02 ± 2.78
*AtPMI2*	48.72 ± 2.05	37.27 ± 2.73	68.45 ± 4.88	79.62 ± 5.94	86.94 ± 1.82	89.74 ± 1.17	29.01 ± 2.68	26.83 ± 4.28	17.01 ± 4.35	31.59 ± 8.73
*OsPMI1*	41.67 ± 1.67	36.36 ± 3.64	74.07 ± 1.76	76.81 ± 5.69	91.33 ± 1.22	91.52 ± 0.90	28.17 ± 0.83	25.80 ± 4.70	23.29 ± 7.20	20.87 ± 5.36

^a^*HPT* in the pCAMBIA 1381 vector was replaced by *EcPMI* or by plant *PMIs*. Then, the transformation of derivative constructs was examined individually.

^b^The ratio of independent resistant calli (events) generated from agrobacteria-incubated calli.

^c^The ratio of independent regenerated events to resistance events.

^d^The ratio of PCR-positive events to total regenerated events.

^e^The ratio of PCR-positive events to agrobacteria-incubated calli.

^f^The ratio of low-copy PCR-positive events to total PCR-positive events; low-copy events (harboring one or two copies) were determined by real-time PCR using a TaqMan probe for the 35S promoter, as described previously. The resistant calli or plants that developed from the same *Agrobacterium* incubation were defined as the same event. All data are presented as the mean ± SD (n = 2).

**Table 3:** Regeneration efficiency and escape rate of *EcPMI* and plant *PMIs* under different Man pressures.

Sugar composition in medium^a^	Regeneration rate (%)				Escape rate (%)^b^			
	*EcPMI*	*CvPMI*	*AtPMI2*	*OsPMI1*	*EcPMI*	*CvPMI*	*AtPMI2*	*OsPMI1*
30 Suc	69.17 ± 4.17	72.50 ± 5.83	65.00 ± 5.00	56.67 ± 3.33	10.81 ± 0.55	10.13 ± 2.63	10.12 ± 1.79	8.68 ± 2.43
10 Man + 20 Suc	17.50 ± 2.50	40.00 ± 6.67	5.00 ± 3.33	5.00 ± 1.67	4.17 ± 4.17	1.79 ± 1.79	0	0
15 Man + 15 Suc	3.33	30.83 ± 5.83	0	0	0	0	–	–
20 Man + 10 Suc	0	17.50 ± 0.83	0	0	–	0	–	–
30 Man	0.83 ± 0.83	12.50 ± 2.50	0	0	0	0	–	–

^a^Suc, sucrose. The numbers preceding Suc and Man indicate the respective sugar concentrations in grams per liter in the regeneration medium. For each *PMI* vector, 60 independent resistance events selected by Man (12.5 g/L Man and 5 g/L Suc) were used to regenerate plants. To compare the effects of sugar compositions, an aliquot of each selection-resistant event (containing 3 well-grown callus nodules) was tested in parallel in the different regeneration media.

^b^The ratio of PCR-negative events to total regenerated events. “–”: not tested. All data are presented as the mean ± SD (n = 2).

**Table 4:** GOI (*HPT* cassette) transformation using plant *PMIs* as SMGs in rice.

SMG^a^	Selection rate (%)	Regeneration rate (%)	Positive rate (%)		Transformation frequency (%)
			SMG^b^	GOI^c^	
*CvPMI*	62.08 ± 4.58	67.98 ± 1.02	90.53 ± 2.22	89.08 ± 0.77	37.67 ± 3.67
*AtPMI2*	52.22 ± 5.56	60.38 ± 10.38	93.68 ± 3.46	93.68 ± 3.46	29.78 ± 7.11
*OsPMI1*	42.86 ± 5.71	61.81 ± 6.43	93.25 ± 1.58	91.86 ± 2.97	24.86 ± 6.57

^a^*Ubq*-driven plant *PMIs* were inserted into a *HPT*-containing pCAMBIA1300 vector individually. Then, the Man-selected transformations of derivative constructs were examined.

^b^The ratio of PCR-positive events for the *Ubq* promoter.

^c^The ratio of PCR-positive events for the *HPT* gene. All data are presented as the mean ± SD (n = 2).
