# Supplementary material for: Plant phosphomannose isomerase as a selectable marker for rice transformation
Source: Sci Rep. 2016 May 13;6:25921. doi: 10.1038/srep25921 (PMC4865823; doi:10.1038/srep25921)
Supplement: Supplementary Information [file srep25921-s1.pdf]

Supplementary information

Plant phosphomannose isomerase as a selectable marker for rice transformation

Lei Hu, Hao Li, Ruiying Qin, Rongfang Xu, Juan Li, Li Li, Pengcheng Wei & Jianbo Yang

Figure S1

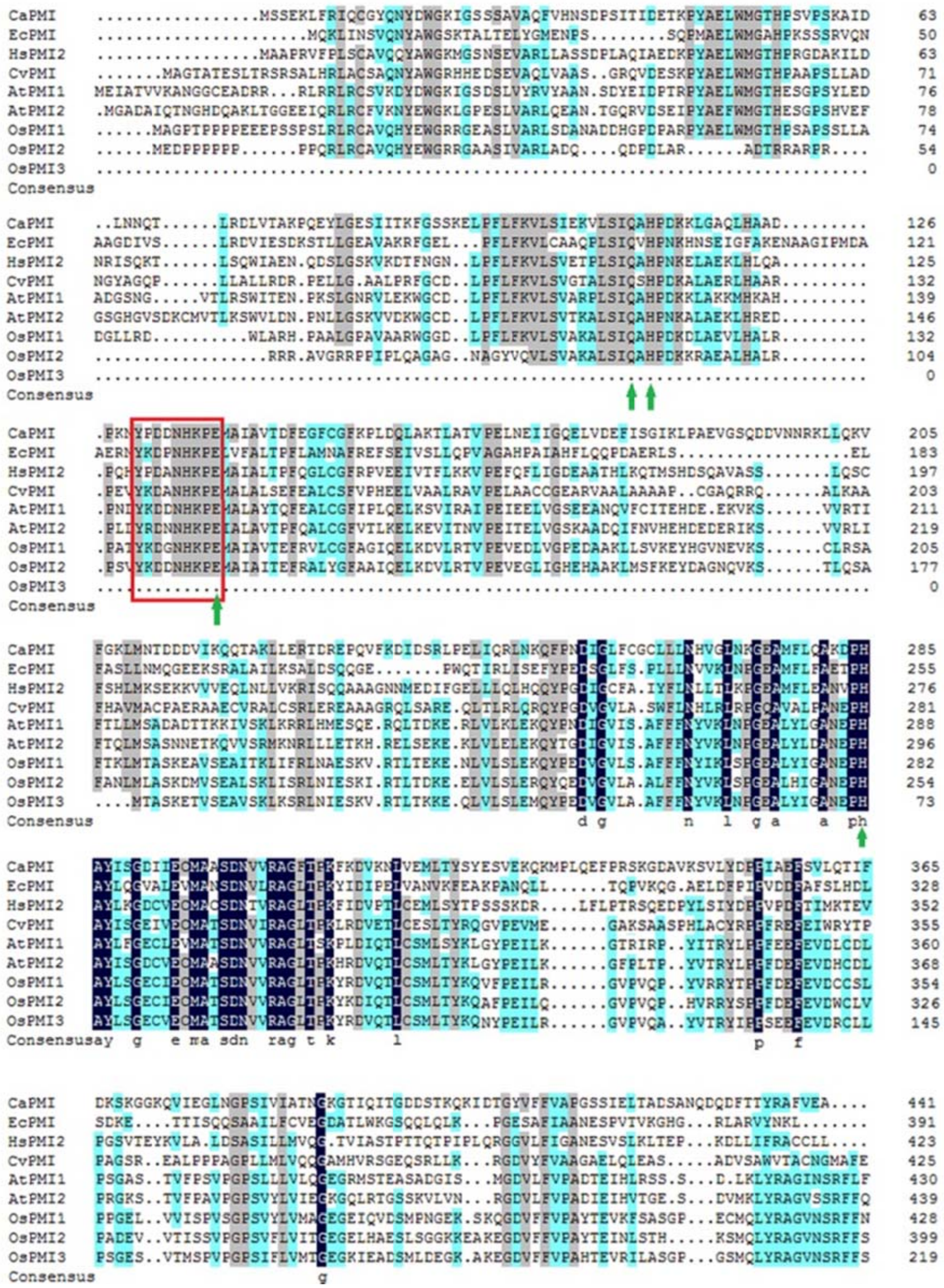

Figure S1. Homology analysis of plant PMI amino acid sequences with typical type I PMIs.

Alignments of the amino acid sequences of the plant PMIs and other PMIs of various species were produced using the BioEdit program. The conserved Y*X*D*X*NHKPE motif is boxed in red. Four zinc-ligand residues, including Gln-111, Glu-138, His-113 and His-285, are labeled with green arrows.

Figure S2

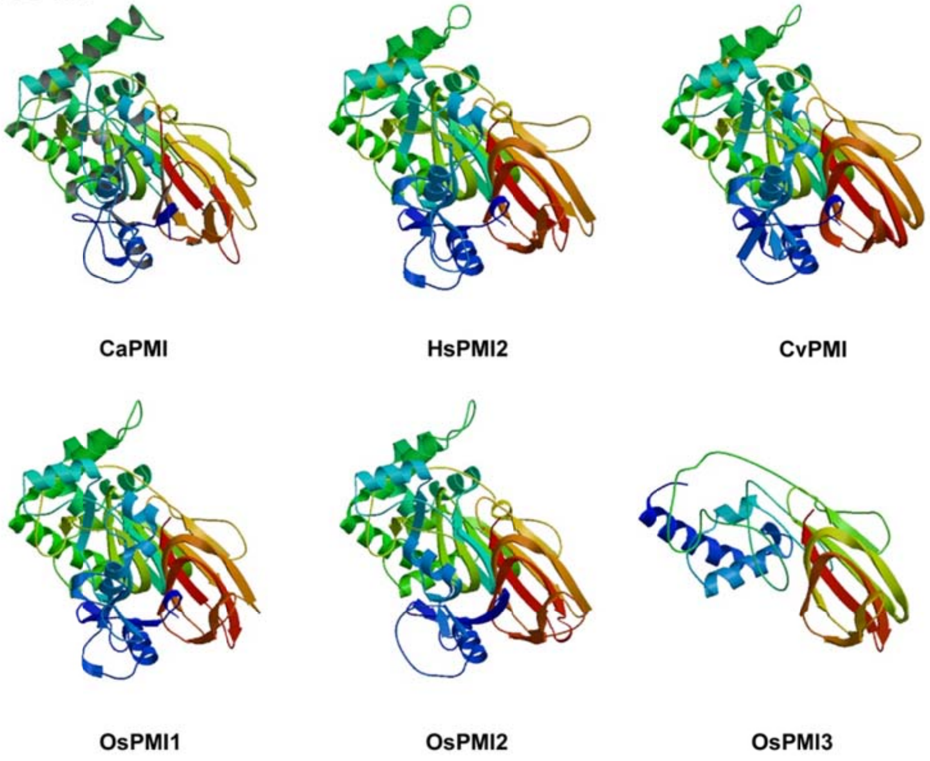

Figure S2. Predicted 3D structures of plant PMIs and typical type I PMIs.
The amino acid sequences were used to predict the 3D structure of the PMIs with a SWISS-MODEL online tool. The images were captured using Cn3D software.

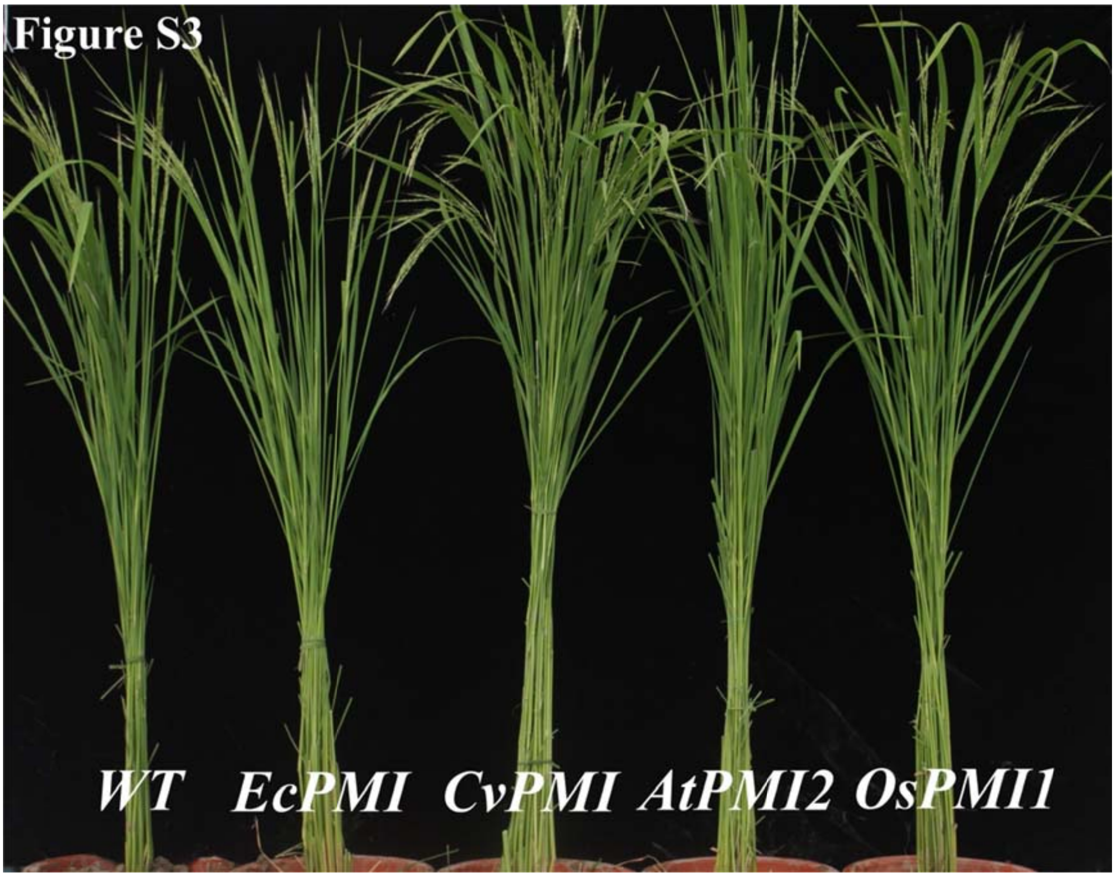

Figure S3. The transgenic Kasalath carrying the *PMI* vectors grew and developed normally in the greenhouse.

The plants were grown for 10 weeks after being transferred to soil. From left to right: the untransformed Kasalath plant, the transgenic Kasalath carrying *EcPMI*, *CvPMI*, *AtPMI2* and *OsPMI1*.

Figure S4

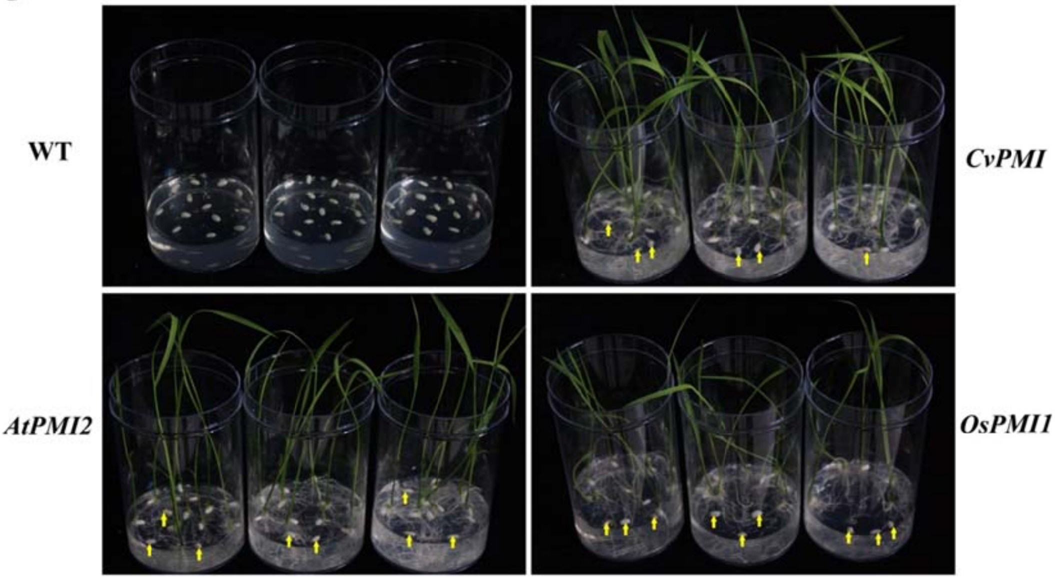

Figure S4. The germination of untransformed Nipponbare and transgenic lines carrying *CvPMI*, *AtPMI2* and *OsPMI1* expression vectors on Man selection medium.

Approximately 15 seeds of untransformed Nipponbare and the transgenic plants expressing *CvPMI*, *AtPMI2* and *OsPMI1* were germinated in MS plus 12.5 g/L Man medium for 10 days. The yellow arrows indicate the failed germination of the transgenic plants.

Table S1. Detailed information regarding the Nipponbare transformations using *EcPMI* and the plant *PMIs* as SMGs.

SMG [a]	Incubated calli [b]		Resistant events [c]		Regenerated events [c]		Positive events [c]		Low-copy events [c,d]	
	Exp.1	Exp.2	Exp.1	Exp.2	Exp.1	Exp.2	Exp.1	Exp.2	Exp.1	Exp.2
EcPMI	250	225	155	145	106	103	96	96	28	24
CvPMI	300	375	190	190	132	144	119	129	44	22
AtPMI2	300	325	140	165	89	121	79	103	10	22
OsPMI1	300	300	120	130	91	94	82	87	25	14

a, The transformation of each *PMI* vector was separately examined in two independent experiments; b, the data represent the number of calli incubated by agrobacteria in each individual experiment; c, the data represent the number of independent events (resistant calli or regenerated plants produced from one original agrobacteria-incubated callus were recognized as an independent event); d, low-copy events (harboring one or two copy) were determined by Real-time PCR by the Taqman probe on the 35S promoter as previously described.

Table S2. Detailed information regarding the Kasalath transformations using *EcPMI* and the plant *PMIs* as SMGs.

SMG [a]	Incubated calli [b]		Resistant events [c]		Regenerated events [c]		Positive events [c]		Low-copy events [c]	
	Exp.1	Exp.2	Exp.1	Exp.2	Exp.1	Exp.2	Exp.1	Exp.2	Exp.1	Exp.2
EcPMI	275	300	135	115	112	97	102	91	20	32
CvPMI	325	325	200	185	171	142	164	131	66	60
AtPMI2	225	275	90	95	77	70	70	62	16	25
OsPMI1	275	200	90	80	64	66	58	61	9	16

a, The transformation of each *PMI* vector was separately examined in two independent experiments; b, the data represent the number of calli incubated by agrobacteria in each individual experiment; c, the data represent the number of independent transformation events.

Table S3. Regeneration of *EcPMI* and the plant *PMIs* selection events under different Man pressures.

Sugar composition in medium [a]		Regeneration events [b]				Positive events [c]			
		EcPMI	*CvPMI*	*AtPMI2*	*OsPMI1*	*EcPMI*	*CvPMI*	*AtPMI2*	*OsPMI1*
30 Suc	Exp.1	44	40	36	36	39	37	33	32
	Exp.2	39	47	42	32	35	41	37	30
10 Man+20 Suc	Exp.1	9	20	1	2	9	20	1	2
	Exp.2	12	28	5	4	11	27	5	4
15 Man+15 Suc	Exp.1	2	15	0	0	2	15	-	-
	Exp.2	2	22	0	0	2	22	-	-
20 Man+10 Suc	Exp.1	0	11	0	0	-	11	-	-
	Exp.2	0	10	0	0	-	10	-	-
30 Man	Exp.1	0	6	0	0	-	6	-	-
	Exp.2	1	9	0	0	1	9	-	-

a, The effect of sugar composition in the regeneration medium were examined in two separate experiments. b, the data represent the number of regenerated events produced from 60 Man-resistant calli; c, the data represent the number of PCR-positive regenerated events. “-“: not tested.

Table S4. GOI transformations using the plant *PMIs* as SMGs in rice.

SMG[a]	Incubated calli [b]	Resistant events [c]	Regenerated events [c]	Positive events [c]		
				SMG [d]	GOI [e]	Both [f]
CvPMI						
Exp.1	200	115	77	68	68	68
Exp.2	150	100	69	64	62	62
AtPMI2						
Exp.1	225	130	92	83	83	83
Exp.2	150	70	35	34	34	34
OsPMI1						
Exp.1	175	85	58	55	55	55
Exp.2	175	65	36	33	32	32

a, The *Ubq*-driven plant *PMIs* were inserted into a *HPT*-contained pCAMBIA1300 vector separately, then the Man-selected transformation of derivative constructs were examined individually; b, the data represent the number of calli incubated by agrobacteria in each individual experiment; c, the data represent the number of independent transformation events; d, positive events determined by sequence specific PCR on the *Ubq* promoter; e, positive events determined by sequence specific PCR on the *HPT* gene; f, the regenerated events positive on both of *Ubq* promoter and *HPT* gene.

Table S5. Segregation of T_1 progenies of the transgenic plants harboring different *PMI-HPT* vectors.

Line	Hygromycin			Man		
	Positive	Negative	Mendelian ratio	Positive	Negative	Mendelian ratio
CvPMI						
1	36	14	3:1	38	12	3:1
2	39	11	3:1	38	12	3:1
3	40	10	3:1	39	11	3:1
4	38	12	3:1	36	13	3:1
5	47	3	15:1	47	3	15:1
6	36	14	3:1	40	10	3:1
AtPMI2						
1	37	13	3:1	36	14	3:1
2	36	14	3:1	36	14	3:1
3	39	11	3:1	35	15	3:1
4	36	14	3:1	38	12	3:1
5	33	17	3:1	35	15	3:1
6	41	9	3:1	40	10	3:1
OsPMI1						
1	38	12	3:1	34	16	3:1
2	50	0	N	50	0	N
3	36	14	3:1	36	14	3:1
4	36	14	3:1	35	15	3:1
5	41	9	3:1	38	12	3:1
6	40	10	3:1	36	14	3:1

3:1 and 15:1 indicate one T-DNA copy and two independent copies, respectively. N suggested the line is not the low-copy line or the segregation of T_1 seeds does not follow a standard Mendelian laws. The chi-square test was used to test goodness-of-fit to the expected segregation ratio.

Table S6. The parallel rice transformation of the *PMI-HPT* vectors using hygromycin selection.

Vector[a]	Incubated calli [b]	Resistant events [c]	Regenerated events [c]	Positive events [c, d]	TFs [e]
CvPMI-HPT					
Exp.1	450	135	32	32	7.11%
Exp.2	450	155	51	51	11.33%
AtPMI2-HPT					
Exp.1	450	125	27	27	6.00%
Exp.2	475	130	44	44	9.26%
OsPMI1-HPT					
Exp.1	425	175	43	43	10.12%
Exp.2	500	145	36	36	7.20%

a, The *Ubq*-driven plant *PMIs* were inserted into a *HPT*-contained pCAMBIA1300 vector separately, then the Hygromycin-selected transformation of derivative constructs were examined individually; b, the data represent the number of calli incubated by agrobacteria in each individual experiment; c, the data represent the number of independent transformation events; d, positive events determined by sequence specific PCR on the *Ubq* promoter; e, The ratio of PCR-positive events to agrobacteria-incubated calli.

Table S7. Primers used in this study.

1. Primers used to amplify the *PMIs*

Primer	Primer sequence (5’ to 3’)
OsPMI1 FP	TTCGCCTCCCTCCTCCCTCCCA
OsPMI1 RP	CACTGACCTTTTACCTACAGCA
OsPMI2 FP:	ATGGAGGACCCGCCGCCGCCGC
OsPMI2 RP:	TTAACTGAAGAATCTGCTGTTT
OsPMI3 FP:	CTTTTACTAATTTAATGACAGC
OsPMI3 RP:	CTCGCAATAACCTTTAATCCCT
CvPMI FP:	ATGGCTGGAACGGCGACAGAGA
CvPMI RP:	CTCAAAGGCCATTCCGTTG
AtPMI2 FP:	ATGGGAGCAGACGCAATCCAAA
AtPMI2 RP:	CAATGTTTGGAAGAATCTGCTG

2. Primers used to construct vectors

Primer	Primer sequence (5’ to 3’, sequences underlined to show endonuclease recognition site)	Restriction site	Experiment
EcPMI-GST FP:	GGATCCATGCAAAAACTCATTAACTCAG	*Bam*HI	GST-EcPMI expression
EcPMI-GST RP:	CTCGAGCAGCTTGTTGTAAACACGC	*Xho*I	
CvPMI-GST FP:	GGATCCATGGCTGGAACGGCGACAGAGA	*Bam*HI	GST-CvPMI expression
CvPMI-GST RP:	CTCGAGCTCAAAGGCCATTCCGTTG	*Xho*I	
AtPMI2-GST FP:	GGATCCATGGGAGCAGACGCAATCCAAA	*Bam*HI	GST-AtPMI2 expression
AtPMI2-GST RP:	CTCGAGCAATGTTTGGAAGAATCTGCTG	*Xho*I	
OsPMI1-GST FP:	GGATCCATGGCCGGCCCTACTCCTCCTC	*Bam*HI	GST-OsPMI1 expression
OsPMI1-GST RP:	CTCGAGATTGAAGAATCTGCTATTGACC	*Xho*I	
OsPMI2-GST FP:	CTCGAGATGGAGGACCCGCCGCCGCCGC	*Xho*I	GST-OsPMI2 expression
OsPMI2-GST RP:	CTCGAGACTGAAGAATCTGCTGTTTACC	*Xho*I	
OsPMI3-GST FP:	GAATTCATGACAGCAAGCAAAGAAACAG	*Bam*HI	GST-OsPMI3 expression
OsPMI3-GST RP:	CTCGAGGCTGAAGAATCTGCTGTTCACC	*Xho*I	
EcPMI-1381 FP:	CTCGAGATGCAAAAACTCATTAACTCAG	*Xho*I	Rice transformation by *EcPMI*
EcPMI-1381 RP:	CTCGAGTTACAGCTTGTTGTAAACACGC	*Xho*I	
CvPMI-1381 FP:	CTCGAGATGGCTGGAACGGCGACAGAGA	*Xho*I	Rice transformation by *CvPMI*
CvPMI-1381 RP:	CTCGAGTCACTCAAAGGCCATTCCGTTG	*Xho*I	
AtPMI2-1381 FP:	CTCGAGATGGGAGCAGACGCAATCCAAA	*Xho*I	Rice transformation by *AtPMI2*
AtPMI2-1381 RP:	CTCGAGCTACAATGTTTGGAAGAATCTG	*Xho*I	
OsPMI1-1381 FP:	CTCGAGATGGCCGGCCCTACTCCTCCTC	*Xho*I	Rice transformation by *OsPMI1*
OsPMI1-1381 RP:	CTCGAGTTAATTGAAGAATCTGCTATTG	*Xho*I	

3. Primers used for semi-quantitative RT-PCR assays

Primer	Primer sequence (5’ to 3’)
ACTIN-SemiF:	TCAGCAACTGGGATGATATGGAG
ACTIN-SemiR:	GCCGTTGTGGTGAATGAGTAAC
EcPMI-SemiF:	TTGACTGAACTTTATGGTATGG
EcPMI-SemiR:	TCCGGCTTGTGGTTAGGATCTT

CvPMI-SemiF:	CGCTCAAAACTATGCCTGGGG
CvPMI-SemiR:	GCTTGTGGTTGGCGTCCTTGTA
AtPMI2-SemiF:	TGGTCACGATCAAGCCAAGT
AtPMI2-SemiR:	CGACAATGCTTTCGTTACTG
OsPMI1-SemiF:	ACGCCGAGCTGTGGATGG
OsPMI1-SemiR:	GGCAAAGCCGCAGAGGAC

4. Primers used for Real-Time PCR assays on the identification of copy numbers

SPS-FP	TCTCCTCGTCCAGTGCTTCTC
SPS-RP	TTGGTGGACGCGCTTCTAG
SPS-Probe	TET-TCCTCGCAACCGAAC-TAM
HPT-FP	CTATTTCTTTGCCCTCGGACGA
HPT-RP	GGACCGATGGCTGTGTAGAAG
HPT-Probe	FAM-CGCCGATAGTGGAAACCGACGCCC-TAM
35S-FP	CGACAGTGGTCCCAAAGA
35S-RP	AAGACGTGGTTGGAACGTCTTC
35S-probe	FAM-TGGACCCCCACCCACGAGGAGCATC-TAM
